# PROM1 and EFTUD2 Expression in High-Grade Clear Cell Renal Cell Carcinoma as a Molecular Marker for Survival Rate

**DOI:** 10.3390/ijms26136296

**Published:** 2025-06-30

**Authors:** Michał Kasperczak, Iga Kołodziejczak-Guglas, Filip Kasperczak, Maciej Wiznerowicz, Andrzej Antczak

**Affiliations:** 1 Department of Urology, J. Struś Hospital in Poznań, Szwajcarska 3, 61-285 Poznan, Poland; filiperkasperczako@gmail.com (F.K.); maciej.wiznerowicz@iimo.pl (M.W.); aa26@poczta.onet.pl (A.A.); 2 International Institute for Molecular Oncology, 60-203 Poznań, Poland; iga.kolodziejczak@iimo.pl; 3 University Hospital of Lord’s Transfiguration, Poznań University of Medical Sciences, 61-848 Poznań, Poland

**Keywords:** PROM1, EFTUD2, clear cell renal carcinoma, proteomics, immunohistochemistry

## Abstract

Clear cell renal cell carcinoma (ccRCC) is a significant global cancer, particularly impacting individuals in Western countries. Despite that, the molecular mechanisms driving renal cell carcinoma progression remain poorly understood, highlighting the need to investigate these mechanisms and identify novel therapeutic targets. Literature evidence suggests that elongation factor Tu GTP binding domain containing 2 (EFTUD2) and prominin (PROM1) gene expression have significant diagnostic potential in early tumor detection, potentially reflecting the trends in progression, and may become a novel therapeutic target. Therefore, this study aimed to evaluate EFTUD2 and PROM1 protein expression on clinical characteristics of ccRCC patients, especially overall and progression-free survival. To achieve that goal, we have combined publicly available liquid chromatography–mass spectrometry (LC-MS/MS) protein expression data with a comprehensive literature review to identify key protein markers for further study and immunohistochemical (IHC) analysis. Our findings highlight the importance of considering protein expression heterogeneity within tumors. The significant variation in EFTUD2 expression, its association with PFS, and its intricate connections with the mRNA splicing machinery underscore the need for a more nuanced understanding of its role in ccRCC. Similarly, the downregulation of PROM1 and its potential effects on cell surface interactions warrant further exploration. Future studies should focus on elucidating the mechanisms underlying these observations, exploring their potential as therapeutic targets, and investigating the specific pathways affected by their dysregulation.

## 1. Introduction

Clear cell renal cell carcinoma (ccRCC), accounting for approximately 3% of global cancer diagnoses, exhibits a high prevalence in Western countries [1,2]. In developed nations, incidental detection via ultrasound, CT, or MRI is the primary mode of diagnosis [3]. The classic triad of hematuria, flank pain, and palpable mass, indicative of renal cell carcinomas (RCC), is observed in only 10% of patients [3]. Representing 75% of all RCCs, ccRCC originates from renal stem cells within the tubular epithelium and proximal nephron [4]. These tumors are prone to hematogenous metastasis, primarily to the liver, lungs, and bones [4]. Inactivation or deletion of the von Hippel–Lindau (VHL) tumor suppressor gene on chromosome 3p is implicated in up to 45% of ccRCC cases [5]. Germline mutations in the VHL gene contribute to 5% of cases (VHL disease), and individuals with tuberous sclerosis gene mutations exhibit an elevated risk of bilateral RCC before age 46 [5]. Modifiable risk factors for RCC include dietary habits, alcohol consumption, obesity, poorly controlled hypertension, smoking, and occupational exposures [6]. Strategies to enhance survival and mitigate disparities emphasize lifestyle modifications, improved healthcare access for underserved populations, and refined imaging guidelines for early detection [7]. Notably, 30% of ccRCC patients present with distant metastasis at diagnosis, resulting in a poor prognosis [8]. The underlying molecular mechanisms of RCC remain incompletely understood, necessitating further investigation to identify novel therapeutic targets.

EFTUD2 (Snu114) forms a stable protein complex and is a constitutive component of the U5 snRNP [9]. This protein-coding gene encoding a spliceosomal GTPase plays a pivotal role in splicing precursor mRNAs (pre-mRNAs) into mature mRNAs [10]. Recent research has revealed that elevated EFTUD2 expression in hepatocellular carcinomas is closely linked to tumor growth and poor survival [11]. This association is substantiated by the observation that EFTUD2 sustains cancer cell viability and enhances the progression of hepatocellular carcinoma through STAT3 activation [12]. Notably, EFTUD2 inhibition has been shown to attenuate the NF-KB signaling pathway’s activation, leading to reduced inflammatory and tumorigenic cytokines in colon cancer [13]. The abovementioned studies imply that EFTUD2 may have additional roles in modulating cancer development, independently or dependently of its canonical function in splicing. Nonetheless, whether EFTUD2 is involved in regulating chemoresistance in different types of cancers, along with the precise molecular mechanisms of its action, remains to be understood.

In turn, prominin-1 (PROM1), also called CD133, is a membrane protein with two bulky extracellular loops with eight glycosylation residues and a ~50 amino acid-long C-terminal tail [14]. As a lipid raft protein, PROM1 is expressed mainly in microvilli or membrane protrusions of various stem cells or progenitor cells [14,15]. PROM1 is a well-known marker of cancer stem cells (CSCs) [16,17,18], a subpopulation of tumor cells that are thought to be drivers of tumor initiation, recurrence, and metastasis [19]. For this reason, PROM1 has been widely studied as a target for cancer therapeutics [16,17,18,20]. In ccRCC, PROM1 demonstrates downregulated expression [21,22]. While PROM1 contributes to cell differentiation in various tissues, its biological role remains unclear [23,24]. In RCC, PROM1 progenitor cells have been observed to differentiate into endothelial cells, promoting vascularization and tumor growth [25,26]. Additionally, its encoding product, CD133, is also downregulated in ccRCC [27]. Although PROM1 serves as a prognostic marker in other cancers like liver and ovarian cancer, its specific functional implications within ccRCC require further investigation [28,29].

This evidence suggests that EFTUD2 and PROM1 gene expression have significant diagnostic potential in early tumor detection, potentially reflecting the trends in progression. However, while previous studies have primarily focused on transcriptomic data or other cancer types, there remains a critical gap in understanding the protein-level expression of these markers in ccRCC and their association with clinical outcomes. Therefore, our study uniquely combines LC-MS/MS proteomics and immunohistochemistry to assess EFTUD2 and PROM1 protein expression in ccRCC, providing novel insights into their prognostic value.

## 2. Results

### 2.1. Protein Expression Values in CPTAC ccRCC

**Figure 1:**
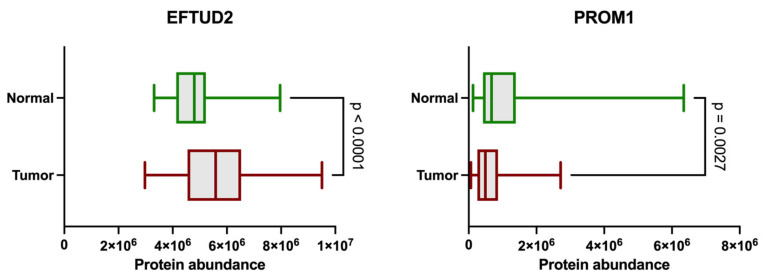
Distribution of protein abundance values for PROM1 (**right**) and EFTUD2 (**left**) between normal adjacent tissue *(n* = 84) and tumor (*n* = 110) samples, as measured by LC-MS/MS. y-axis represent sample groups (Normal and Tumor) and x-axis indicate protein abundance (log2-transformed intensities). Green boxes represent normal adjacent tissue samples, and red boxes denote tumor samples. For EFTUD2, *p* < 0.0001, and for PROM1, *p* = 0.0027.

Among the proteins analyzed, EFTUD2 showed lower abundance in ccRCC tissues compared to NATs (Figure 1). Conversely, PROM1 exhibited lower levels in tumor samples compared to NATs. These differences were statistically significant (*p* < 0.0001 for EFTUD2 and *p* = 0.0027 for PROM1), as determined by the Mann–Whitney U test. Interestingly, we observed a substantial spread in EFTUD2 expression within tumor samples, with some specimens showing extremely high, and others extremely low, levels of this protein.

### 2.2. Immunohistochemical Analysis of EFTUD2 and PROM1

**Figure 2:**
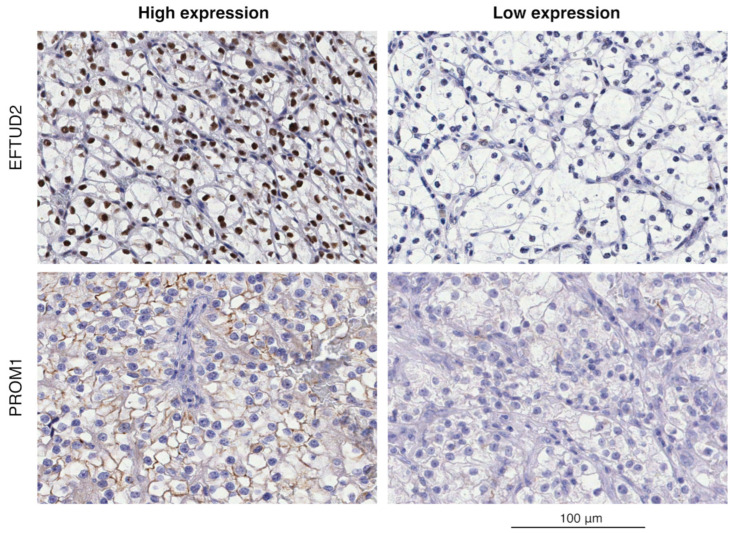
Immunohistochemical analysis showing immunostaining patterns in representative tumor sections for EFTUD2 and PROM1 high and low expression in the ccRCC validation cohort. Brown color indicates positive immunoreaction. Expression levels are based on staining intensity and evaluated by H-score. Scale bar = 100 μm.

Immunohistochemical analysis from the ccRCC validation cohort revealed that EFTUD2 expression is predominantly within the nuclei of tumor cells, while PROM1 is located in the cytoplasm (Figure 2). Many cells show some level of positivity with heterogeneous staining intensity.

### 2.3. EFTUD2 and PROM1 Protein Expression as Measured by IHC

**Figure 3:**
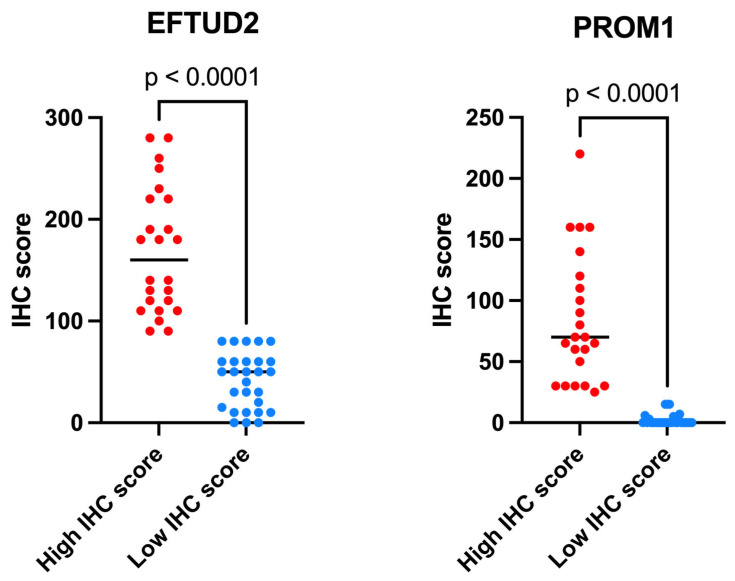
The detailed breakdown of the high and low EFTUD2 and PROM1 IHC score groups. The expression of both proteins shows statistically significant differences (*p* < 0.0001) between high and low score groups.

For the analyzed validation cohort, samples were categorized into “high score” and “low score” groups based on their obtained IHC scores for EFTUD2 and PROM1 expression. Figure 3 provides a detailed breakdown of these expression groups. The results confirm the findings from the CPTAC protein expression analysis, demonstrating multiple instances of both very high and very low protein expression in the analyzed tumor samples. EFTUD2 generally presented higher expression in the analyzed samples, along with a significant spread of scores within the high IHC score group. The low IHC score groups exhibited a smaller spread of scores and fewer notable outliers for both proteins.

### 2.4. EFTUD2 and PROM1 Protein Expression Correlated with Patients’ Clinical Outcomes

**Figure 4:**
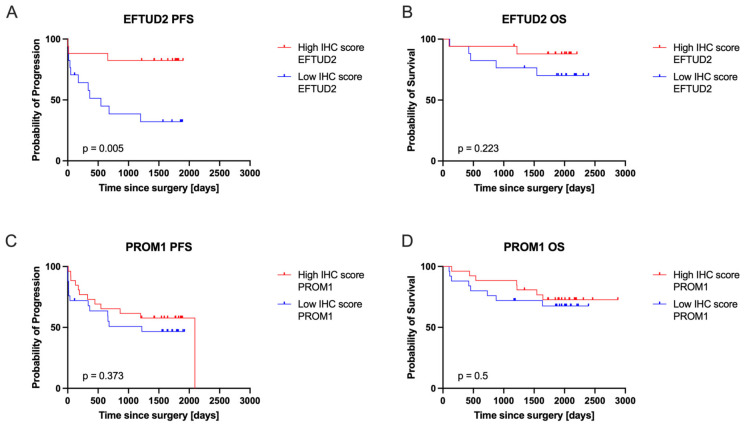
Kaplan–Meier survival curves comparing overall survival (OS) (**B**) between high and low EFTUD2 IHC score groups in ccRCC patients in the validation cohort; (**D**) between high and low PROM1 IHC score groups in ccRCC patients in the validation cohort; and progression-free survival (PFS) (**A**) between high and low EFTUD2 IHC score groups in ccRCC patients in the validation cohort; (**C**) between high and low PROM1 IHC score groups in ccRCC patients in the validation cohort. Groups were defined based on protein-specific H-score cutoffs calculated as the average H-score across all samples (EFTUD2 cutoff = 90; PROM1 cutoff = 25; see Section 4 Methods and Table 2). *p*-values from the log-rank test are presented in the bottom-left corner of each graph.

To assess the clinical relevance of EFTUD2 and PROM1 protein expression in ccRCC patients, we compared OS and PFS between the high and low IHC score groups. The results of the analysis are presented in Figure 4. The only significant difference (*p* = 0.005) was detected in PFS between high and low EFTUD2 expression groups. Patients with low EFTUD2 IHC scores showed worse PFS compared to patients with high EFTUD2 IHC scores. This difference was associated with a 5.016 (95% CI 1.737–14.49) Cox hazard ratio for the low EFTUD2 IHC score group.

### 2.5. Identification of the Top Significantly Enriched Gene Ontologies Associated with EFTUD2 and PROM1

**Figure 5:**
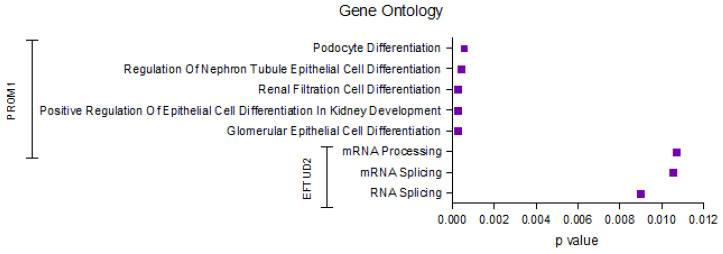
The results of Gene Ontology enrichment analysis for PROM1 and EFTUD2. The y-axis lists all significantly enriched GO terms, and the x-axis represents the corresponding *p*-values. Terms were identified based on statistical significance in enrichment analysis and reflect biological processes associated with each protein.

The results of GO analysis (Figure 5) indicate a significant enrichment of ontologies related to kidney development and mRNA processing. Specifically, PROM1 is associated with several Gene Ontology terms related to podocyte and epithelial cell differentiation in the kidney. EFTUD2, on the other hand, shows a significant association with mRNA processing and splicing terms. These findings suggest distinct roles of PROM1 and EFTUD2 in kidney development and mRNA metabolism, respectively.

### 2.6. Protein–Protein Interaction Networks Reveal Key Associations of EFTUD2 and PROM1 at the Protein Level

**Figure 6:**
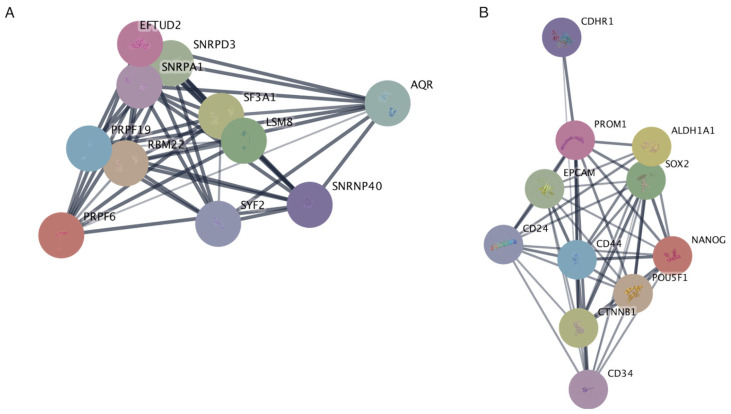
The results of protein–protein interaction (PPI) analysis for EFTUD2 (**A**) and PROM1 (**B**), performed in Cytoscape based on STRING database data. Each node (colored ball) represents a protein, with a 3D structural preview displayed inside the node. Edges (connecting lines) represent predicted or known protein–protein interactions.

The protein–protein interaction analysis for EFTUD2 highlights a cluster of genes primarily associated with mRNA processing and splicing (Figure 6A). The PPI network reveals associations among EFTUD2, SNRPD3, SNRPA1, SF3A1, LSM8, and SNRNP40, which have been previously implicated in mRNA processing and splicing. Furthermore, the analysis for PROM1 revealed a cluster associated with stemness and cell adhesion (Figure 6B). Genes such as PROM1, ALDH1A1, EPCAM, SOX2, NANOG, and POU5F1 form a highly interconnected cluster in the PPI network and are commonly associated with stem cell maintenance and differentiation. Additionally, the presence of genes like CDH1, CD24, CD44, and CD34 may suggest involvement in cell adhesion and signaling within this network.

## 3. Discussion

This study investigated the expression of EFTUD2 and PROM1 in ccRCC and their potential as prognostic markers. Our analysis of the CPTAC ccRCC dataset revealed significant differences in the abundance of these proteins between tumor tissues and NATs. EFTUD2 was significantly upregulated in ccRCC, suggesting a potential involvement in tumor progression, while PROM1 was downregulated. The wide range of EFTUD2 expression in tumor samples, from extremely high to extremely low, was further corroborated by IHC analysis, indicating heterogeneity within ccRCC.

IHC staining provided further insights into the cellular localization of these proteins. Specifically, we found that EFTUD2 expression is predominantly within the nuclei of tumor cells. This localization is consistent with our analysis showing that EFTUD2 interacts with numerous nuclear proteins involved in mRNA splicing, including core spliceosome components (SNRPD3, SNRPA1), proteins essential for snRNP formation (SF3A1, LSM8, SNRNP40), and factors involved in spliceosome assembly, catalysis, and regulation (PRPF19, RBM22, AQR, PRPF6). Notably, EFTUD2 also interacts with SYF2, linking cell cycle regulation to splicing. This diverse interactome underscores the complexity of EFTUD2’s role in RNA processing, suggesting that its expression levels could influence splicing fidelity and efficiency, with potential downstream effects on gene expression and cellular function [30].

Our GO enrichment analysis further suggests its involvement in mRNA splicing. It is plausible that disruptions in EFTUD2-mediated splicing contribute to the dysregulation of gene expression programs critical for tumor suppression, leading to a more aggressive phenotype and poorer clinical outcomes. Crucially, correlating protein expression with clinical outcomes revealed that low EFTUD2 IHC scores were significantly associated with worse PFS in ccRCC patients. This is particularly interesting given the observed variability in EFTUD2 expression within tumor samples, suggesting that while EFTUD2 is generally upregulated in tumors, its reduced expression might indicate a more aggressive disease. This hypothesis is supported by studies in other cancers, such as colorectal cancer, where EFTUD2 has been shown to influence c-MYC stability and contribute to chemoresistance [31].

Beyond its role in mRNA splicing, EFTUD2 appears to have broader functions in cancer development. It has been implicated in modulating NF-κB signaling [31], influencing cell viability and migration [32], and regulating the innate macrophage immune response [33], ultimately serving as a potential biomarker in different cancer types [10,34]. These diverse roles highlight the complex and context-dependent nature of EFTUD2 in cancer. For example, in endometrial cancer, EFTUD2 expression correlated with poor prognosis and was linked to increased expression of RIG-I, suggesting an interplay between EFTUD2 and the tumor microenvironment [35]. Furthermore, research in hepatocellular carcinoma has revealed a connection between EFTUD2 and metabolic reprogramming, where EFTUD2 regulates the protein stability of YTHDF3, an m6A “reader” protein involved in RNA metabolism [36]. Finally, EFTUD2 may be used as a therapeutic strategy, as identified by Sato et al., where its knockdown in breast cancer cells led to an increase in apoptotic cells [37].

PROM1 (CD133) was primarily found on the cell membrane, in line with its role as a transmembrane stem cell marker [38]. This localization allows PROM1 to interact with various cell surface proteins involved in diverse cellular processes. Our results show that PROM1 interacts with cadherins like CDHR1 and cell adhesion molecules like EpCAM, potentially influencing cell–cell adhesion and tissue integrity. Its interaction with ALDH1A1 could influence cellular differentiation and response to oxidative stress. Furthermore, it interacts with key stem cell regulators (e.g., SOX2, NANOG, POU5F1), suggesting a role in stem cell maintenance and differentiation, and with molecules like CD24, CD44, and CTNNB1, potentially affecting cell adhesion, migration, and signaling. The interaction with CD34, a hematopoietic stem cell marker, could be relevant in the context of cancer stem cells (CSC). These interactions highlight the complex role of PROM1 in cellular communication and function. Indeed, our GO enrichment analysis further showed that PROM1 appears to be associated with kidney development and epithelial cell differentiation, supporting its multifaceted involvement in both normal tissue development and potentially aberrant processes in cancer.

While our study found it to be downregulated in ccRCC compared to NAT without showing any significant correlation to the patients’ clinical condition, some studies have associated high PROM1 expression with poor prognosis, [39,40]. This discrepancy might be explained by the diverse roles PROM1 plays in different cellular contexts. For instance, PROM1 can be expressed on both stem cells and differentiated cells, and its glycosylation status can vary with cellular differentiation and malignant transformation [41,42,43]. Further highlighting PROM1’s complex role, different studies reveal that PROM1’s prognostic significance varies across different cancer types, with its expression and methylation having contrasting implications in gliomas and papillary RCC [44]. Additionally, research in thyroid cancer has shown that its expression varies across different subtypes and that a specific gene signature, including high PROM1 expression, correlates with worse recurrence-free survival [27]. Furthermore, the localization of PROM1 within the cell may influence its function, with cytoplasmic expression potentially being associated with increased tumor aggressiveness [45]. This complexity underscores the challenges in identifying and characterizing CSCs in ccRCC, as single marker expression may not accurately reflect the CSC population [46]. Indeed, some studies suggest that PROM1+ cells in ccRCC may represent a heterogeneous population [43]. This heterogeneity has significant implications for understanding ccRCC progression and treatment response. If PROM1+ cells have varying degrees of stemness and differentiation, therapies targeting PROM1 may have unpredictable effects.

Despite these challenges, PROM1 holds potential as a biomarker in ccRCC. Studies have explored its use in predicting metastasis and recurrence, warranting further investigation [27]. Interestingly, targeting specific pathways in PROM1+ CSCs can influence their survival and sensitivity to therapy. For example, activating TNFR2 signaling promotes the proliferation of PROM1+ CSCs in ccRCC and increases their sensitivity to cell cycle-dependent cytotoxicity [47]. In line with the complex role of PROM1 in ccRCC, its involvement in other cancers also appears to be context dependent. For example, in ovarian cancer, PROM1 overexpression has been linked to poor prognosis and worse response to treatment, particularly in patients with TP53 mutations [48].

These findings, along with our observation of EFTUD2’s association with PFS in ccRCC, emphasize the importance of considering these proteins’ diverse roles and interactions in cancer development. Our study contributes to a growing body of knowledge highlighting the importance of protein–protein interaction and network perturbations in tumor progression [49], underscoring the need for further investigation into the complex interplay of these factors in ccRCC. However, interpretations of the results should be cautious, as the study’s modest sample size and focused design, while intentional for exploratory comparisons, suggest that these findings are preliminary and necessitate validation in larger, independent cohorts. The absence of formal multiple testing correction further underscores the preliminary nature of these findings and the need for validation in larger studies employing appropriate statistical methods.

This work further underscores the emerging significance of non-traditional biomarkers and molecular features in predicting RCC outcomes. Unlike conventional clinical or imaging parameters, which often provide a static snapshot, these molecular indicators offer deeper, more dynamic insights into tumor biology. Our investigation into the protein expression patterns and interactions of EFTUD2 and PROM1 aligns with this evolving landscape, where understanding the nuanced molecular underpinnings of ccRCC is paramount. These non-traditional markers encompass a diverse range of molecular characteristics, such as specific protein expression patterns, post-translational modifications, and intricate protein–protein interaction networks within tissues [50,51,52]. Moreover, important examples also include those detectable in easily accessible biological samples like blood or urine, such as circulating tumor DNA (ctDNA), circulating tumor cells (CTCs), and microRNAs (miRNAs) [29,53,54]. The integration of these advanced molecular insights is crucial for moving beyond broad disease classifications to achieve more precise prognostic and predictive models for individual patients [55]. By capturing real-time disease progression and tumor heterogeneity, these markers can significantly refine risk stratification and guide personalized therapeutic approaches, ultimately aiming to improve clinical decision-making and patient outcomes [56]. Furthermore, considering the significant clinical relevance of metastasectomy and its impact on survival, particularly in carefully selected patients with oligometastatic disease where complete surgical removal of metastatic lesions can lead to prolonged survival in renal cell carcinoma, some reflection in this direction would situate the study in a broader clinical context [57,58]. While our study focuses on initial prognostic markers, the utility of such markers could extend to informing patient selection for metastasectomy, potentially refining patient management strategies and improving overall survival in those with oligometastatic disease [58].

Our study has several limitations that should be acknowledged. Firstly, our analysis is primarily based on correlational data. Further mechanistic studies are needed to fully substantiate our findings and elucidate the precise molecular mechanisms by which EFTUD2 and PROM1 influence ccRCC progression. Secondly, our study focused on profiling EFTUD2 and PROM1 expression, limiting the scope of our investigation. We did not include a comprehensive analysis of clinical/histological progression, a critical aspect of ccRCC, nor did we examine the role of these proteins in the context of therapies such as chemotherapy, which is relevant for advanced-stage disease. While we leveraged CPTAC’s proteomic data, future studies could integrate its genomic information for deeper mechanistic insights, expanding upon our current findings. Additionally, due to our limited sample size, we could not perform stratified analyses by variables like histological grade or age group. While such analyses would offer valuable subgroup-specific insights into metastatic risk, our current cohort is underpowered for this. Additionally, our study lacks in vitro or in vivo experimental validation, which will be essential to confirm the functional relevance of our findings in a biological context. Future studies should investigate the complex interplay between EFTUD2/PROM1 expression, clinical/histological progression, and therapeutic responses in ccRCC to provide a more complete understanding of their roles in this disease, and larger cohorts are needed to enable robust stratification.

## 4. Materials and Methods

### 4.1. Patients and Cohorts

**Figure 7:**
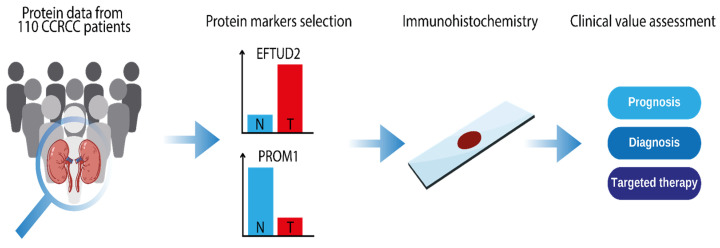
Study design overview: The study involved the acquisition of protein data from the CPTAC discovery ccRCC cohort dataset and subsequent analysis of protein expression of tumor (T, *n* = 110) and normal adjacent tissue (*N*, *n* = 84) to select candidate protein markers. The selected protein markers were then used as a target of immunohistochemical analysis. Finally, we have conducted an assessment of the clinical value of the selected proteins for prognosis, diagnosis, and targeted therapy for ccRCC patients.

Our study utilized a ccRCC discovery cohort from the Clinical Proteomic Tumor Analysis Consortium (CPTAC), which included 110 untreated ccRCC cases and 84 matched normal adjacent tissue (NAT) samples, as described by Clark et al. [30]. We integrated publicly available LC-MS/MS protein expression data (referred to as protein abundance, which are log2-transformed intensities) with a comprehensive literature review to identify key protein markers for subsequent investigation and immunohistochemical (IHC) analysis (Figure 7).

The proteomic data from the CPTAC dataset allowed for a thorough examination of protein markers involved in ccRCC development. Based on their biological relevance, we selected proteins exhibiting differential expression in tumors compared to normal adjacent tissues: EFTUD2 (higher expression) and PROM1 (lower expression). The expression of these validated markers was then linked to clinical data via IHC analysis. This integrated approach, combining proteomic data acquisition, marker validation, and clinical correlation, enhances our understanding of ccRCC biology, progression, and potential clinical implications.

The validation cohort consisted of 52 ccRCC samples from individuals aged 31 to 84 years, with 23 of these participants also part of the CPTAC discovery study. Detailed demographic and clinical information, including age, gender, race, grade, and stage, was collected. Over a five-year follow-up period, 26 patients developed metastasis, while 26 did not. This study focused on adult patients with histopathologically confirmed ccRCC tumors. Ethical approval was obtained in accordance with CPTAC guidelines. Patients who had received systemic treatment or were diagnosed with other cancers in the previous 12 months were excluded from the study.

### 4.2. Immunohistochemistry and Pathology Evaluation

IHC staining was performed using the Dako Autostainer Link 48, with preprogrammed staining protocols and the EnVision visualizing kit (Cat No. K800221-2, Dako, Agilent Technologies Inc., Carpinteria, CA, USA). This polymer-based system directly conjugates HRP to the secondary antibody complex, eliminating the need for a separate secondary antibody. The reaction was visualized using 3,3′-diaminobenzidine (DAB) as the chromogen.

Tissue samples were collected retrospectively, fixed in 10% neutral buffered formalin for 24 h, and then embedded in paraffin. Following this, 4-micron sections were cut from these formalin-fixed paraffin-embedded (FFPE) blocks and placed on positively charged slides. Antigen retrieval was carried out using Dako Target Retrieval Solution, High pH (Cat No. S2367), in a PT Link Pre-Treatment Module (Dako, Agilent Technologies Inc.) at 97 °C for 20 min. After antigen retrieval, sections were incubated with primary antibodies for 30 min at room temperature. Two primary antibodies were used for the clinical validation cohort, as detailed in Table 1.

The IHC-stained slides were independently assessed by three pathologists using the H-score method. The H-score is a semi-quantitative method that considers both staining intensity and the percentage of positive cells, resulting in a score from 0 to 300. The equation used for H-score calculation is as follows:

H-score = (% of cells stained at intensity 1 × 1) + (% of cells stained at intensity 2 × 2) + (% of cells stained at intensity 3 × 3)

The elements of this formula are represented as follows:

- Intensity 1 indicates weak staining;
- Intensity 2 indicates moderate staining;
- Intensity 3 indicates strong staining.

These percentages represent the proportion of cells at each staining intensity. The H-score, derived by summing the products of intensity scores and their corresponding percentages, provides a comprehensive assessment of both the quantity and intensity of IHC staining in a specimen [31].

A total of 52 ccRCC FFPE samples were used to evaluate PROM1 and EFTUD2 expression through IHC analysis. These samples were categorized into groups for further analysis based on their H-scores. Patients with IHC scores above the cutoff value were placed in the “High IHC score” group, while those with scores below the cutoff were placed in the “Low IHC score” group (Table 2). The cutoff value for each protein was determined by calculating the average of all H-scores from the 52 IHC slides. For EFTUD2, the cutoff was set at an H-score of 90; samples with H-scores ≥ 90 were assigned to the High IHC score group. For PROM1, the cutoff was established at an H-score of 25, with scores ≥ categorized as High IHC expression.

### 4.3. Digital Image Acquisition and Archiving

Digital scans of all IHC slides were generated at 20× magnification using a ScanScope AT Turbo whole slide scanner (Aperio/Leica Microsystems, Vista, CA, USA). These digital images, saved in .svs format, were subsequently reviewed by expert pathologists using ImageScope software (version 12.3.3, Aperio, Vista, CA, USA) for detailed assessment. Access to the digitized images was provided via a password-protected Synology Rack Station server (RS18017xs+).

### 4.4. Statistical Analysis

Differences in protein abundance between normal and tumor tissues in ccRCC were computed using a non-parametric Wilcoxon rank-sum test. For comparing IHC scores between the High IHC score and Low IHC score groups of the validation cohort, the Wilcoxon rank-sum test was also performed. Additionally, differences in protein abundance between groups were assessed using the Mann–Whitney U test.

Overall survival (OS) and progression-free survival (PFS) analyses for the validation cohort were conducted by dividing the validation cohort into two groups (High IHC score and Low IHC score, separately for each protein target). These groups were then compared using the Kaplan–Meier method and the log-rank (Mantel–Cox) test. Hazard ratios (HR) and their 95% confidence intervals (CI) were calculated to assess the impact on survival outcomes. All data were analyzed using GraphPad Prism 10 software. A *p*-value < 0.05 was considered statistically significant for all analyses.

### 4.5. Gene Ontology Enrichment Analysis

To gain deeper insights into the biological processes and tumor mechanisms associated with PROM1 and EFTUD2, we conducted a Gene Ontology (GO) enrichment analysis. This analysis aimed to identify the most significant Reactome pathways enriched for each protein, thereby elucidating their functional implications in ccRCC.

Gene Ontology enrichment analysis for EFTUD2 and PROM1 was conducted using the Enrichr platform (available at https://maayanlab.cloud/Enrichr (accessed on 13 April 2025)), a comprehensive and interactive web-based tool for gene list enrichment analysis. GO terms were evaluated across the Biological Process (BP) category. Enrichr computes enrichment by combining statistical significance from Fisher’s exact test with a correction for multiple hypothesis testing. The output includes GO terms ranked by adjusted *p*-values and combined scores, enabling identification of overrepresented biological annotations associated with the input genes. A statistical significance threshold of *p* < 0.05 was applied to determine the biological processes, ensuring that the identified processes were highly likely to be biologically relevant [32,33,34].

### 4.6. Protein–Protein Interaction as Determined by String Database

Protein–protein interaction (PPI) networks for EFTUD2 and PROM1 were constructed using the stringApp for STRING database (version 2.1.1) integrated with Cytoscape (version 3.10). Individual analyses were conducted for each protein to identify their interaction protein partners. STRING was queried with the official gene symbols “EFTUD2” and “PROM1,” and interactions were retrieved based on high-confidence scores (e.g., confidence score ≥ 0.8). The network construction included both direct (physical) and indirect (functional) associations, limited to interactions supported by experimental evidence or curated databases. The resulting PPI networks were visualized and analyzed in Cytoscape, showing only statistically significant results (*p* < 0.05). Node sizes and edge thickness were adjusted to reflect the number of interactions and confidence levels, respectively.

## 5. Conclusions

Our findings highlight the importance of considering protein expression heterogeneity within tumors. The significant variation in EFTUD2 expression, its association with PFS, and its intricate connections with the mRNA splicing machinery underscore the need for a more nuanced understanding of its role in ccRCC. Similarly, the downregulation of PROM1 and its potential effects on cell surface interactions warrant further exploration. Future studies should focus on elucidating the mechanisms underlying these observations, exploring their potential as therapeutic targets, and investigating the specific pathways affected by their dysregulation.

## Figures and Tables

**Table 1:** Primary antibodies used for immunohistochemistry in this study.

Antibody	Source	Identifier	Clone	Dilution
Rabbit polyclonal anti-EFTUD2	Atlas Antibodies	Cat# HPA022021, RRID:AB_1848032	Polyclonal	1:400
Rabbit polyclonal anti-PROM1	Atlas Antibodies	Cat# HPA004922, RRID:AB_1846238	Polyclonal	1:250

**Table 2:** Classification of IHC specimen based on H-scores.

Protein	H-Score Range—High IHC Score Group	H-Score Range—Low IHC Score Group	Reaction
EFTUD2	90–280, *n* = 24	10–80, *n* = 28	Nuclear
PROM1	25–220, *n* = 24	0–15, *n* = 28	Cytoplasmic, membranous

## Data Availability

All data on which the manuscript was based, if not already included in its text, are available from the corresponding author upon reasonable request.

## References

[1] Ferlay J., Colombet M., Soerjomataram I., Dyba T., Randi G., Bettio M., Gavin A., Visser O., Bray F. (2018). Cancer incidence and mortality patterns in Europe: Estimates for 40 countries and 25 major cancers in 2018. Eur. J. Cancer.

[2] Capitanio U., Bensalah K., Bex A., Boorjian S.A., Bray F., Coleman J., Gore J.L., Sun M., Wood C., Russo P. (2019). Epidemiology of Renal Cell Carcinoma. Eur. Urol..

[3] DeCastro G.J., McKiernan J.M. (2008). Epidemiology, Clinical Staging, and Presentation of Renal Cell Carcinoma. Urol. Clin. N. Am..

[4] Chevrier S., Levine J.H., Zanotelli V.R.T., Silina K., Schulz D., Bacac M., Ries C.H., Ailles L., Jewett M.A.S., Moch H. (2017). An Immune Atlas of Clear Cell Renal Cell Carcinoma. Cell.

[5] Creighton C.J., Hernandez-Herrera A., Jacobsen A., Levine D.A., Mankoo P., Schultz N., Du Y., Zhang Y., Larsson E., Sheridan R. (2012). Integrated analyses of microRNAs demonstrate their widespread influence on gene expression in high-grade serous ovarian carcinoma. PLoS ONE.

[6] Chow W.H., Dong L.M., Devesa S.S. (2010). Epidemiology and risk factors for kidney cancer. Urology.

[7] Howlader N., Noone A.M., Krapcho M., Miller D., Brest A., Yu M., Ruhl J., Tatalovich Z., Mariotto A., Lewis D.R. (2019). SEER Cancer Statistics Review, 1975–2016.

[8] Capitanio U., Montorsi F. (2016). Renal cancer. Lancet.

[9] Achsel T., Ahrens K., Brahms H., Teigelkamp S., Lührmann R. (1998). The Human U5-220kD Protein (hPrp8) Forms a Stable RNA-Free Complex with Several U5-Specific Proteins, Including an RNA Unwindase, a Homologue of Ribosomal Elongation Factor EF-2, and a Novel WD-40 Protein. Mol. Cell Biol..

[10] Wood K.A., Eadsforth M.A., Newman W.G., O’Keefe R.T. (2021). The Role of the U5 snRNP in Genetic Disorders and Cancer. Front. Genet..

[11] Lv C., Li X.J., Hao L.X., Zhang S., Song Z., Ji X.D., Gong B. (2022). Over-activation of EFTUD2 correlates with tumor propagation and poor survival outcomes in hepatocellular carcinoma. Clin. Transl. Oncol..

[12] Tu M., He L., You Y., Li J., Yao N., Qu C., Huang W., Xu L., Luo R., Hong J. (2020). EFTUD2 maintains the survival of tumor cells and promotes hepatocellular carcinoma progression via the activation of STAT3. Cell Death Dis..

[13] Lv Z., Wang Z., Luo L., Chen Y., Han G., Wang R., Xiao H., Li X., Hou C., Feng J. (2019). Spliceosome protein Eftud2 promotes colitis-associated tumorigenesis by modulating inflammatory response of macrophage. Mucosal Immunol.

[14] Weigmann A., Corbeil D., Hellwig A., Huttner W.B. (1997). Prominin, a novel microvilli-specific polytopic membrane protein of the apical surface of epithelial cells, is targeted to plasmalemmal protrusions of non-epithelial cells. Proc. Natl. Acad. Sci. USA.

[15] Röper K., Corbeil D., Huttner W.B. (2000). Retention of prominin in microvilli reveals distinct cholesterol-based lipid micro-domains in the apical plasma membrane. Nat. Cell Biol..

[16] Liu G., Yuan X., Zeng Z., Tunici P., Ng H., Abdulkadir I.R., Lu L., Irvin D., Black K.L., Yu J.S. (2006). Analysis of gene expression and chemoresistance of CD133+ cancer stem cells in glioblastoma. Mol. Cancer.

[17] Bao Bao S., Wu Q., McLendon R.E., Hao Y., Shi Q., Hjelmeland A.B., Dewhirst M.W., Bigner D.D., Rich J.N. (2006). Glioma stem cells promote radioresistance by preferential activation of the DNA damage response. Nature.

[18] Florek M., Haase M., Marzesco A.M., Freund D., Ehninger G., Huttner W.B., Corbeil D. (2005). Prominin-1/CD133, a neural and hematopoietic stem cell marker, is expressed in adult human differentiated cells and certain types of kidney cancer. Cell Tissue Res..

[19] Ricci-Vitiani L., Lombardi D.G., Pilozzi E., Biffoni M., Todaro M., Peschle C., De Maria R. (2007). Identification and expansion of human colon-cancer-initiating cells. Nature.

[20] Yin S., Li J., Hu C., Chen X., Yao M., Yan M., Jiang G., Ge C., Xie H., Wan D. (2007). CD133 positive hepatocellular carcinoma cells possess high capacity for tumorigenicity. Int. J. Cancer.

[21] Wang J., Yuan L., Liu X., Wang G., Zhu Y., Qian K., Xiao Y., Wang X. (2018). Bioinformatics and functional analyses of key genes and pathways in human clear cell renal cell carcinoma. Oncol. Lett..

[22] D’Alterio C., Cindolo L., Portella L., Polimeno M., Consales C., Riccio A., Cioffi M., Franco R., Chiodini P., Cartenì G. (2010). Differential role of CD133 and CXCR4 in renal cell carcinoma. Cell Cycle.

[23] Sagrinati C., Netti G.S., Mazzinghi B., Lazzeri E., Liotta F., Frosali F., Ronconi E., Meini C., Gacci M., Squecco R. (2006). Isolation and characterization of multipotent progenitor cells from the Bowman’s capsule of adult human kidneys. J. Am. Soc. Nephrol..

[24] Ronconi E., Sagrinati C., Angelotti M.L., Lazzeri E., Mazzinghi B., Ballerini L., Parente E., Becherucci F., Gacci M., Carini M. (2009). Regeneration of glomerular podocytes by human renal progenitors. J. Am. Soc. Nephrol..

[25] Bussolati B., Bruno S., Grange C., Buttiglieri S., Deregibus M.C., Cantino D., Camussi G. (2005). Isolation of Renal Progenitor Cells from Adult Human Kidney. Am. J. Pathol..

[26] Bussolati B., Grange C., Collino F., Graziano M.E., Ferrando U., Camussi G. (2006). CD133+ Renal Progenitor Cells Contribute to Tumor Angiogenesis. Am. J. Pathol..

[27] Kim K., Ihm H., Ro J.Y., Cho Y.M. (2011). High-level expression of stem cell marker CD133 in clear cell renal cell carcinoma with favorable prognosis. Oncol. Lett..

[28] Zhou L., Yu K.H., Wong T.L., Zhang Z., Chan C.H., Loong J.H., Che N., Yu H.J., Tan K.V., Tong M. (2022). Lineage tracing and single-cell analysis reveal proliferative Prom1+ tumour-propagating cells and their dynamic cellular transition during liver cancer progression. Gut.

[29] Dansonka-Mieszkowska A., Szafron L.A., Kulesza M., Stachurska A., Leszczynski P., Tomczyk-Szatkowska A., Sobiczewski P., Parada J., Kulinczak M., Moes-Sosnowska J. (2022). PROM1, CXCL8, RUNX1, NAV1 and TP73 genes as independent markers predictive of prognosis or response to treatment in two cohorts of high-grade serous ovarian cancer patients. PLoS ONE.

[30] Clark D.J., Dhanasekaran S.M., Petralia F., Pan J., Song X., Hu Y., da Veiga Leprevost F., Reva B., Lih T.-S.M., Chang H.-Y. (2019). Integrated Proteogenomic Characterization of Clear Cell Renal Cell Carcinoma. Cell.

[31] Hirsch F.R., Varella-Garcia M., Bunn P.A., Di Maria M.V., Veve R., Bremmes R.M., Barón A.E., Zeng C., Franklin W.A. (2003). Epidermal growth factor receptor in non-small-cell lung carcinomas: Correlation between gene copy number and protein expression and impact on prognosis. J. Clin. Oncol..

[32] Xie Z., Bailey A., Kuleshov M.V., Clarke D.J.B., Evangelista J.E., Jenkins S.L., Lachmann A., Wojciechowicz M.L., Kropiwnicki E., Jagodnik K.M. (2021). Gene Set Knowledge Discovery with Enrichr. Curr. Protoc..

[33] Chen E.Y., Tan C.M., Kou Y., Duan Q., Wang Z., Meirelles G.V., Clark N.R., Ma’ayan A. (2013). Enrichr: Interactive and collaborative HTML5 gene list enrichment analysis tool. BMC Bioinform..

[34] Kuleshov M.V., Jones M.R., Rouillard A.D., Fernandez N.F., Duan Q., Wang Z., Koplev S., Jenkins S.L., Jagodnik K.M., Lachmann A. (2016). Enrichr: A comprehensive gene set enrichment analysis web server 2016 update. Nucleic Acids Res..

[35] Zhu X., Li C., Gao Y., Zhang Q., Wang T., Zhou H., Bu F., Chen J., Mao X., He Y. (2024). The feedback loop of EFTUD2/c-MYC impedes chemotherapeutic efficacy by enhancing EFTUD2 transcription and stabilizing c-MYC protein in colorectal cancer. J. Exp. Clin. Cancer Res..

[36] Ma H., Suo L., Zhao J., Ma R., Wang Q., Liu J., Qiao J., Wu J., An J., Liu Y. (2024). Prognostic biomarkers based on GUF1, EFTUD2 and GSPT1 targets affecting migration of gastric cancer cells. Transl. Cancer Res..

[37] Chen F., Wang Q., Zhou Y. (2021). The construction and validation of an RNA binding protein-related prognostic model for bladder cancer. BMC Cancer.

[38] Zhou W., Chen Y., Luo R., Li Z., Jiang G., Ou X. (2021). Identification of Biomarkers Related to Immune Cell Infiltration in Hepatocellular Carcinoma Using Gene Co-Expression Network. Pathol. Oncol. Res..

[39] Zhang Z.G., Shi Z.D., Dong J.J., Chen Y.A., Cao M.Y., Li Y.T., Ma W.M., Hao L., Pang K., Zhou J.H. (2023). Novel potential urinary biomarkers for effective diagnosis and prognostic evaluation of high-grade bladder cancer. Transl. Cancer Res..

[40] Beyer S., Müller L., Mitter S., Keilmann L., Meister S., Buschmann C., Kraus F., Topalov N.E., Czogalla B., Trillsch F. (2022). High RIG-I and EFTUD2 expression predicts poor survival in endometrial cancer. J. Cancer Res. Clin. Oncol..

[41] Zhou R., Ni W., Qin C., Zhou Y., Li Y., Huo J., Bian L., Zhou A., Li J. (2022). A functional loop between YTH domain family protein YTHDF3 mediated m6A modification and phosphofructokinase PFKL in glycolysis of hepatocellular carcinoma. J. Exp. Clin. Cancer Res..

[42] Sato N., Maeda M., Sugiyama M., Ito S., Hyodo T., Masuda A., Tsunoda N., Kokuryo T., Hamaguchi M., Nagino M. (2015). Inhibition of SNW1 association with spliceosomal proteins promotes apoptosis in breast cancer cells. Cancer Med..

[43] Bahn M.S., Ko Y.G. (2023). PROM1-mediated cell signal transduction in cancer stem cells and hepatocytes. BMB Rep..

[44] da Costa W.H., Rocha R.M., da Cunha I.W., da Fonseca F.P., Guimaraes G.C., de Cassio Zequi S. (2012). CD133 immunohistochemical expression predicts progression and cancer-related death in renal cell carcinoma. World J. Urol..

[45] Yin A.H., Miraglia S., Zanjani E.D., Almeida-Porada G., Ogawa M., Leary A.G., Olweus J., Kearney J., Buck D.W. (1997). AC133, a Novel Marker for Human Hematopoietic Stem and Progenitor Cells. Blood.

[46] Bussolati B., Collino F., Camussi G. (2012). CD133+ cells as a therapeutic target for kidney diseases. Expert. Opin. Ther. Targets.

[47] Wang H., Wang X., Xu L., Zhang J., Cao H. (2021). A pan-cancer perspective analysis reveals the opposite prognostic significance of CD133 in lower grade glioma and papillary renal cell carcinoma. Sci. Prog..

[48] Beck A.C., Rajan A., Landers S., Kelley S., Bellizzi A.M., Lal G., Sugg S.L., Howe J.R., Chan C.H., Weigel R.J. (2022). Expression of cancer stem cell markers in tall cell variant papillary thyroid cancer identifies a molecular profile predictive of recurrence in classic papillary thyroid cancer. Surgery.

[49] Saeednejad Zanjani L., Madjd Z., Abolhasani M., Andersson Y., Rasti A., Shariftabrizi A., Asgari M. (2017). Cytoplasmic expression of CD133 stemness marker is associated with tumor aggressiveness in clear cell renal cell carcinoma. Exp. Mol. Pathol..

[50] Hasmim M., Bruno S., Azzi S., Gallerne C., Michel J.G., Chiabotto G., Lecoz V., Romei C., Spaggiari G.M., Pezzolo A. (2015). Isolation and characterization of renal cancer stem cells from patient-derived xenografts. Oncotarget.

[51] Feng G., Jiang F., Pan C., Pu C., Huang H., Li G. (2014). Quantification of peripheral blood CD133 mRNA in identifying metastasis and in predicting recurrence of patients with clear cell renal cell carcinoma. Urol. Oncol. Semin. Orig. Investig..

[52] Bradley J.R., Wang J., Pacey S., Warren A.Y., Pober J.S., Al-Lamki R.S. (2020). Tumor necrosis factor receptor-2 signaling pathways promote survival of cancer stem-like CD133+ cells in clear cell renal carcinoma. FASEB Bioadv..

[53] Ferrari I., De Grossi F., Lai G., Oliveto S., Deroma G., Biffo S., Manfrini N. (2024). CancerHubs: A systematic data mining and elaboration approach for identifying novel cancer-related protein interaction hubs. Brief. Bioinform..

[54] Graham J., Heng D.Y.C., Brugarolas J., Vaishampayan U. (2018). Personalized Management of Advanced Kidney Cancer. Am Soc Clin Oncol Educ Book.

[55] Saliby R.M., Saad E., Kashima S., Schoenfeld D.A., Braun D.A. (2024). Update on Biomarkers in Renal Cell Carcinoma. Am. Soc. Clin. Oncol. Educ. Book.

[56] Laru L., Ronkainen H., Ohtonen P., Vaarala M.H. (2024). The impact of metastasectomy on survival of patients with synchronous metastatic renal cell cancer in Finland: A nationwide study. Scand. J. Surg..

[57] Ferriero M., Cacciatore L., Ochoa M., Mastroianni R., Tuderti G., Costantini M., Anceschi U., Misuraca L., Brassetti A., Guaglianone S. (2023). The Impact of Metastasectomy on Survival Outcomes of Renal Cell Carcinoma: A 10-Year Single Center Experience. Cancers.

[58] Oki R., Takemura K., Urasaki T., Fujiwara R., Numao N., Yonese J., Miura Y., Yuasa T. (2025). Prevailing challenges in personalized treatment for metastatic renal cell carcinoma: A narrative review. Expert Rev. Anticancer Ther..

